# Preparedness, Adaptation, and Innovation: Approach to the COVID-19 Pandemic at a Decentralized, Quaternary Care Department of Emergency Medicine

**DOI:** 10.5811/westjem.2020.8.48624

**Published:** 2020-09-25

**Authors:** Anna Q. Yaffee, Elizabeth Peacock, Roslyn Seitz, George Hughes, Philip Haun, Michael Ross, Tim P. Moran, Andrew Pendley, Nataisia Terry, David W. Wright

**Affiliations:** Emory University, Department of Emergency Medicine, Atlanta, Georgia

## Abstract

The COVID-19 pandemic has required healthcare systems to be creative and adaptable in response to an unprecedented crisis. Below we describe how we prepared for and adapted to this pandemic at our decentralized, quaternary-care department of emergency medicine, with specific recommendations from our experience. We discuss our longstanding history of institutional preparedness, as well as adaptations in triage, staffing, workflow, and communications. We also discuss innovation through working with industry on solutions in personal protective equipment, as well as telemedicine and methods for improving morale. These preparedness and response solutions and recommendations may be useful moving forward as we transition between response and recovery in this pandemic as well as future pandemics.

## BACKGROUND

The COVID-19 pandemic has led to over one million infections in the United States (US), making the US the leader in both total number of infections and deaths due to SARS-CoV-2. Variation in public health response around the world is one of the many reasons that resulted in some countries being affected more heavily by the pandemic than others. Countries such as Vietnam and South Korea, which had early case counts^1^ but aggressive countermeasures such as shelter-in-place orders and widely available testing had success against the virus. Efficacy of a response is partially related to preparedness.

The most serious recent pandemic the US experienced was H1N1 influenza in 1918.^2^ Several pandemics since then, including H1N1 influenza in 2009, were impactful and led to preparedness plans at institutional, state, and national levels.^3^ The Ebola virus disease (EVD) outbreak in West Africa in 2014–2015 led to additional preparedness ventures in the US.^4^ This included standing up the National Ebola Training and Education Center (NETEC), recently rebranded as the National Emerging Special Pathogen Training and Education Center, to assist with frontline and facility-level preparedness focused on pathogens like Ebola transmitted through body fluid exposure.^5^ Conversely, some countries in Asia have had prior experience with respiratory pathogens through outbreaks of severe acute respiratory syndrome (SARS) and Middle East respiratory syndrome and have a robust response plan.^6^–^8^ We know this preparedness is important, as swift response by countries such as South Korea led to disproportionately fewer deaths due to COVID-19 than others affected at the same time.^1^

As of this writing (August 18, 2020), the State of Georgia ranked fourth in the US in numbers of confirmed COVID cases (238,861),^9^ and has experienced 4727 deaths.^10^ The Emory University Healthcare system is a large, decentralized, quaternary care system with multiple hospitals and many levels of medical and nursing leadership. The Emory Department of Emergency Medicine covers the entire system within a single leadership structure representing over 500,000 annual patient visits across seven emergency departments (ED). Our experience with COVID-19 has been within this context, and in our initial wave we saw over 1500 COVID-19 positive patients in our EDs (March 14 – April 27, 2020). Although we have not experienced the incredible surges seen in New York, our case burden necessitated a robust response, and we have had many successes as well as opportunities to improve our care and processes. For example, testing shortages statewide initially reduced our ability to test. In response, our institution worked to stand up our own proprietary testing, only to be later plagued by swab shortages. We believe that an assessment of our pandemic response, preparedness, and implementation provides an opportunity to reflect and share our experiences with others in the medical community. Below we detail the main take-aways and recommendations from our experience.

## PREPAREDNESS DURING PRE-PANDEMIC TIMES IS IMPERATIVE

Because of our institution’s pre-existing relationship with national public health leadership including faculty who hold joint appointments with the CDC, we were fortunate to have a robust, serious, communicable disease program in a steady state of preparedness. This state of preparedness was born out of a mission to provide assistance to employees of the CDC, physically located beside our campus in Atlanta, and bolstered by the EVD outbreak in 2014–15 in which the program successfully and safely cared for multiple patients with EVD. This program includes both nurses and providers trained and ready to care for patients with serious communicable diseases in our biocontainment unit. Regular training and drills involve personal protective equipment (PPE) donning and doffing sessions for powered air-purifying respirators (PAPR) level and high-level (face mask or N95, eye protection, contact gown, and gloves) PPE, a group of nursing “super users” who practice delivering care in their PPE quarterly, and real-time exercises in the ED alongside our Serious Communicable Diseases Unit (SCDU) team.

A real asset has been our ED’s close association with nurses and faculty members embedded within the SCDU team, the benefit of which became readily apparent when the ED was included early in planning as COVID-19 became a reality in the US. These pre-existing skills were helpful as we had established practices converting our ED to care for a serious communicable disease, and had been early adopters of a universal travel screen to isolate patients with infectious symptoms away from our large population of immunocompromised patients. We also had high-level PPE trainers ready to mobilize as it became necessary to partner with ancillary staff, such as radiology and environmental services. At one ED, we trained 218 nurses and providers to safely don and doff high-level PPE in one week using the scalable, pre-trained “super user” approach. Although other institutions may not have the same relationship with the CDC, there are still many ways that an institutional state of preparedness can be maintained.

### Preparedness Recommendations

1. Maintain a cadre of healthcare workers trained as PAPR and high-level PPE super users, including nurses and providers, with quarterly recertification and donning and doffing drills in PPE.
2. Maintain a standard operating procedure (SOP) for care for patients with serious communicable disease, with which department administration is familiar and can be rapidly deployed and scaled as necessary.
3. Use a universal travel screen at patient entry points to screen and isolate patients with infectious symptoms.^11^

## ADAPTABILITY – IN TRIAGE, WORKFLOW, STAFFING, AND COMMUNICATION

**Figure 1:**
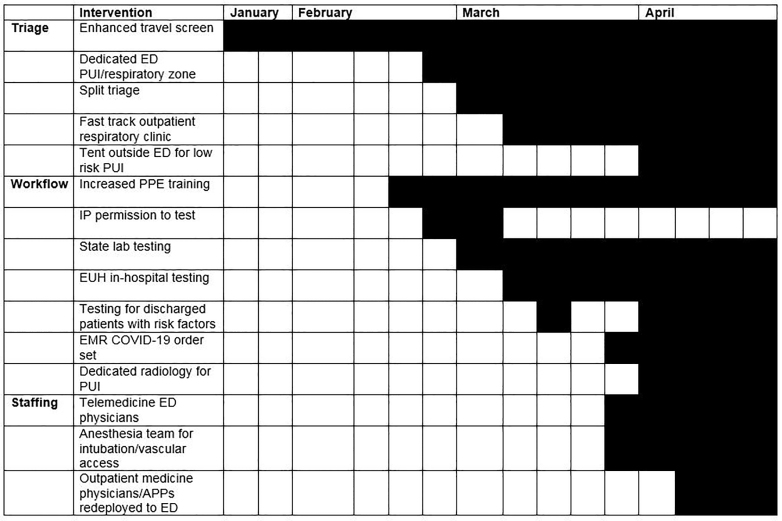
Timeline of key interventions in the Emergency Department (ED) at Emory University Hospital (EUH) during the COVID-19 response (January 13, 2020 – April 27, 2020). *PUI*, person under investigation; *IP*, infection prevention; *EMR*, electronic medical record.

Our department was able to adapt to the rapidly evolving information regarding the science, availability of resources, and system responses in addressing the changing needs of our patients, as well as to hone early, less-than-ideal processes. Some of this stems from the baseline adaptive outlook of emergency medicine (EM) operations, where the constant state of changing workflow truly is our steady state.^12^ Below we detail specific adaptations in *one of our EDs*, including our triage, workflow, and staffing algorithms (Figure 1). These adaptations, protocols and practices were widely adapted across our EM service line.

### Triage

We started to screen patients with recent travel to China with fever or cough in January 2020, guided by early recommendations from our Infection Prevention (IP) team. Patients were triaged to one of two negative pressure rooms and IP was contacted for co-management of each patient. Patients pending triage waited in a small, enclosed, negative-airflow waiting room, which was ultimately found not to be ideal as patients with fever and respiratory complaints were sitting in close proximity for hours; thus, early on we adapted triage procedures to manage the increase in volume of persons under investigation (PUI) for COVID-19, specifically through a split-flow operational model. Because of our immunocompromised patient population and small physical space, we split our triage and ED flow into infectious/respiratory complaints and non-respiratory complaints before the patients entered the treatment space. We initially used a symptom screen to identify infectious/respiratory complaints that included fever, cough, and shortness of breath, and then expanded this screen when additional characteristic COVID-19 symptoms were recognized. This split triage model enabled flexibility and kept infectious respiratory patients physically apart from the rest of our patient population, including immunocompromised patients without infectious complaints.

The tiered triage model was based on risk assessment through a revision of the 2009 H1N1 SORT criteria,^13^ revised to the known presenting signs, symptoms, and risk factors for patients with COVID-19 from literature out of China and the US.^14^–^17^ Triage now occurs physically outside the ED by a triage nurse, behind a screen and in PPE, based on medical history, symptoms, heart rate, and oxygen saturation. Low-risk PUIs are directed to a rapid discharge area (initially a physically separate, fast-track area, and subsequently transitioned to a tent outside the ED) with COVID-specific discharge and home-isolation instructions and goal arrival-to-discharge time of 30 minutes. This protocol became the standard applied across our system’s EDs. We created minimalist protocolized work-ups including chest radiograph (CXR) and nasopharyngeal swab, managed by advanced practice providers (APP). A room was dedicated for chest CXRs for patients likely to be discharged. Intermediate-risk patients identified based on comorbidities and social situation (chronic lung, heart or kidney disease; immunocompromised; diabetes; communal housing) waited in the respiratory-patient waiting room later to be triaged by APP/telemedicine doctors into the rapid discharge area or into the main respiratory zone. High-risk patients based on clinical signs were brought immediately back into the respiratory zone for physician evaluation.

Initial challenges with this process included the physical layout of the ED, which required modification with temporary walls and markings delineating warm zones to prevent crossover of infectious vs noninfectious patients. Other challenges included adapting and flexing the model based on upticks in patient volumes and acuity. All cardiac arrest and stroke patients were triaged as PUI into a resuscitation bay, with all staff wearing high-level PPE (N95 respirator, face shield or eye protection, gown, and gloves). Our physical space was also modified to adapt to this changing triage and flow, including addition of high-efficiency particulate air filtration and temporary walls to delineate the respiratory zone.

#### Triage Recommendations

1. Institute a split triage and flow model to separate infectious/respiratory vs noninfectious complaints, using a tiered triage approach based on comorbidities, clinical condition, and infectious symptoms.
2. Modify physical space as needed to maintain discrete infectious and non-infectious zones.

### Workflow

During the first three months of the developing COVID-19 pandemic, our diagnostic testing ability changed due to testing modifications and fluctuating availability of supplies. We had a unified testing strategy across institution and agreed upon at the administration level, which was adapted as needed in conjunction with IP. Initially, we used state health department tests and called IP for permission to test. Subsequently, we began to use an Emory-developed RT-PCR test, which reduced turn-around time to <24 hrs (Figure 1). At a system-level, a fast-track outpatient respiratory clinic was developed to redirect flow of low-risk patients from the ED. When the supply chain for swabs was interrupted, we preserved testing for those patients being admitted, and eventually were able to test all admitted patients for cohorting and infection control while in hospital. As our availability of swabs increased, we expanded testing for patients being discharged with moderate to severe risk factors as well as healthcare workers. We also began to deploy the Cepheid rapid test in cases where early knowledge of the results could aid with disposition, such as clearing patients to return to communal living (nursing homes, shelters, or other close quarters). We also used the rapid test to send respiratory patients with negative test results and alternative diagnosis to the clinical decision unit, and prior to providing positive pressure ventilation and respiratory treatments in the ED. Use of the rapid test decreased our ED boarding pending test results for these certain special populations, and otherwise admitted patients were usually not held in the ED for results.

Within our practice, we made significant changes in workflow. We implemented protocols to reduce spread of the virus and for patient and staff safety, including temporarily stopping the use of noninvasive positive pressure ventilation and nebulizers in the ED. We also intubated using video-assisted laryngoscopy in conjunction with plastic drapes or shields. We collaborated with ancillary departments to create more efficient workflow protocols with radiology, laboratory, and environmental services to conserve PPE, expedite PUI exams, and provide more timely diagnostic results. Our CT scanner decontamination protocol was streamlined to require that hospital-grade sanitizer be used to wipe it clean after masked patients.

Many of our colleagues from various specialties assisted in offloading non-PUI patient volume from the ED, such as dental pain and orthopedic injuries, which were quickly rerouted after an appropriate medical screening exam to be seen by oral surgery and orthopedic surgery off-site. With encouragement from hospital administration via incident command working groups, subspecialties were able to shift their practice to ensure rapid access to care for their patients to offset the need for ED referral for evaluation, and our psychiatry program created a mechanism to streamline psychiatric patient boarding and placement. An anesthesia team was put together to perform intubations as well as arterial and central line placement to free emergency physicians to care for other critically ill patients while conserving PPE. We also modified the electronic health record for COVID-19 orders to facilitate ordering of labs, imaging, and isolation precautions.

#### Workflow Recommendations

1. Unify and streamline testing strategy across institution, to prioritize limited testing capacity for those patients for whom the test result would have the greatest impact on their care or disposition.
2. Consider implementation of personnel-protective safeguards, particularly during aerosol-generating procedures, such as use of evidence-based shields, video laryngoscopy, and avoidance of positive pressure ventilation and nebulization.
3. Use other services to streamline and offset workload for emergency providers, including alternate areas for patient care, rapid clinic follow-up, and proceduralists to assist as needed in the ED.

### Staffing

This flexible triage and patient care model led to modifications to our ED staffing. In the pre-pandemic steady state, we had already implemented a seasonal, influenza-surge staffing model to include an overnight on-call emergency physician to care for patients admitted to the intensive care unit but boarding in the ED. We quickly adapted this existing surge staffing for increased respiratory patient volume. When the volume ebbed, presumably due to stay-at-home precautions, we flexed providers off the schedule while maintaining pay to increase wellness, morale, and prepare for future anticipated surges. Additionally, when providers needed to come off the schedule for illness, our process enabled us to preserve the on-call system by flexing in providers from the surge schedule to fill available shifts. Daily needs assessments of staffing occurred, enabling this flexible model to activate providers onto the schedule as needed. The development of telemedicine, discussed further below, enabled us to be more flexible with rounding in our observation units to enable the ED providers on shift to focus on higher acuity care. At the system level, 22 outpatient internal and family medicine attending physician and APP volunteers were trained in ED operations and PPE early on in the pandemic. These colleagues were deployed to the ED to cover lower-acuity patients in the non-respiratory zone and for aftercare responsibilities, freeing emergency physicians for higher acuity cases.

#### Staffing Recommendations

1. Implement a surge staffing schedule to enable as needed flexing physicians and APPs on and off the schedule to address ED surge as well as fill in for providers who need to come off the schedule for illness.
2. Consider credentialing family and internal medicine physicians and APPs, to offload lower-acuity workload from emergency providers as needed.

### Communication - in one place, among all stakeholders

As the pandemic impacts our healthcare system, email traffic has increased, including communications from many sources such as the healthcare system, department leads, and individual hospital sites. Providers and staff reported information overload from the sheer volume of emails as well as the quickly changing guidelines and operating procedures in response to new information on the pandemic as well as supply chain challenges. We surveyed our providers about how they felt about communications and found that of the 71 respondents out of 240 physicians and APPs surveyed, 42% felt they were receiving the right amount of information from the institution, while 46% felt they were receiving too much information from the institution. Sixty-four percent of physicians and 68% of APPs surveyed felt that they were receiving the right amount of information from our ED and the medical directors of their sites.

The main areas of concern regarding communication and clinical work included provider safety and frequently changing protocols. In response to this feedback, we moved toward developing a living, web-based, SOP document, which was updated frequently and served as a central source of up-to-date information. This allowed us to avoid minor email updates and enable providers and staff to have one central repository of information for protocols and safety. The SOP also included a built-in feedback form that provided feedback directly to the creator. Between March 29 – April 22, 2020, the SOP underwent nine iterations. Institutionally, we moved toward one daily update email. Additionally, the ED medical directors began holding weekly meetings on a virtual platform, which served to update the clinical group regarding operation changes, brainstorming for solutions, and as formal processing group sessions for debriefing of personal and professional stressors related to the pandemic. Finally, for situational awareness, the chair of EM provided a weekly podcast to keep faculty, staff, and residents up-to-date on the latest changes and ongoing system-level initiatives.

Early in our response to the pandemic, it was recognized that physician and nursing communications were occurring in a siloed fashion, thus resulting in ineffective process implementation as well as frustrations across both disciplines. These communications were then coordinated and centralized to occur within the incident command center (ICC) structure outlined below as well as with pre-shift huddles between charge nurse and hand-off physicians to determine real-time plans for the day. This informed the rapid coordination of workflows and modification of clinical protocols within the ED by including all key stakeholders in a daily meeting where tasks were assigned and coordinated through project managers. Ultimately, this coordination enabled us to push forward many of our initiatives.

We also began to improve coordination as a system with an ICC structure. ED operations was identified as a workgroup within the ICC structure. This workgroup managed ED operations with daily meetings between all medical directors across hospitals as well as separate ICC meetings. These changes enabled us to be unified as a system and communicate as one voice at the system level. As our department covers a number of hospitals with different leadership structures and policies, these daily meetings across hospitals were important to ensure that our SOPs functioned appropriately across each site and that best practices were shared and quickly disseminated across our EDs.

#### Communication Recommendations

1. Streamline and standardize multiple levels of communication between department and institution via an incident command structure with EM represented in the ICC structure.
2. Coordinate communication between physician and nursing leadership.
3. Create a SOP document that is readily accessible and updated regularly for providers and staff to access centralized information.

## INNOVATION

### Working with Industry

Faced with the potential healthcare surge of COVID-19 patients as well as potential for PPE shortages and sick providers, we worked toward innovative solutions to mitigate these risks. Our close relationships between other academic institutions and industries helped with creative solutions to PPE supply issues, including development of 3-D printed faceshields and novel intubation plexiglass shields^18^ in coordination with the Georgia Institute of Technology. Other PPE solutions included investigation and trialing of respirator sterilization and reuse strategies, such as ultraviolet (UV) sterilization. Finally, many solutions were primarily technology-based, including a mobile, web-based application, C19check.com, to provide the general public a source of information to assess their risk of severe COVID-19 disease and what to do next to help mitigate a hospital surge.^19^ This application, translated into multiple languages to maximize impact, assisted our general patient population with decision-making as to when to come to the ED, and was developed out of an established relationship between industry and our institution. The application was promoted through university channels online as well as to the public through university media relations in order to raise awareness of the checker and facilitate guidance to the public. The application also has the capability to expedite ED triage process by providing an option for patients to self-triage with the application. Patients can then show the triage provider their output, as a provider-hands-free option, to help sort the patients into their triage risk category, thus theoretically facilitating social distancing even within the ED.

#### Industry Recommendations

1. Identify outstanding needs (eg, PPE) and institutional partners with skillsets to fill these needs.
2. Consider leveraging novel or existing web-based technology to inform the general public and facilitate healthcare utilization.

### Telemedicine

We also developed an EM telemedicine initiative, which was identified as a tool that could be deployed to help with challenges such as access to care, resource optimization, physician safety, and a mechanism to allow quarantined but asymptomatic providers to contribute clinically. We obtained tablets mounted on rolling stands, which could facilitate easy transition between patients, for a telemedicine physician located in a central office location outside the ED. This system included a telemedicine stethoscope so the physician could virtually examine the patient as needed. Initially, we sought to deploy the telemedicine physician as a way to evaluate and treat low-risk respiratory patients in our tent external to the ED; however, we quickly learned the physical layout, acoustics, and visual limitations of the tent made telemedicine use in this way unfeasible. Our telemedicine program has since been successfully deployed at multiple different stages along the patient care continuum, thereby expanding its utility. From a prehospital perspective, patients who do not require emergent care are seen by clinic physicians using telemedicine. This has improved the patient care experience while mitigating ED resource use and staff exposure. In ED triage, the telemedicine emergency physician is connected with a triage nurse to provide rapid medical evaluation and input initial orders. For low-acuity patients, the nurse in the room assists the telemedicine emergency physician with a full evaluation, including facilitating telemedicine stethoscope use, and can complete the entire work-up and discharge plan.

The telemedicine physician has been used to staff APPs when needed in the ED, and to round remotely in each hospital’s ED observation unit. One physician has been able to simultaneously care for low-acuity patients in observation units as well as respiratory patient triage areas in two hospitals at once, thus optimizing workflows, improving patient flow, and reducing PPE consumption. Finally, telemedicine has been used for patient follow-up, including COVID-19 test results sent during an ED visit or high-risk patients not tested during their index visit. All patients have follow-up using an algorithm that involves escalation as indicated from a nurse call, to a physician or APP call, to a telemedicine visit or to a COVID-19 clinic visit, or finally to return to the ED. Moving forward, additional opportunities for telemedicine include pre-emergency medical services for evaluation by providers to determine need for transport or for saturated departments as a way to continue management of stabilized patients.

#### Telemedicine Recommendations

1. Consider implementation of a telemedicine program for ease of prehospital triage, to streamline low-acuity emergency patient care, or for patient follow-up.

### Morale

Our department has a strong institutional focus on wellness during steady state including wellness initiatives and a funding stream.^20^–^22^ This baseline has easily translated into initiatives during the COVID-19 pandemic including a focus on health, safety, and wellness of faculty and staff. In terms of health and safety, before known local community transmission of SARS-CoV-2, we fast-tracked staff testing through a systemwide COVID-19 hotline. We had early clarification of the process of caring for our own, enabling return to work, and developed strategies to maintain salaries. We also prioritized learner safety, removing medical students and off-service rotating residents from the ED early on in the pandemic, and encouraged EM residents to see only non-PUI patients initially until our safety procedures solidified. Scribes were also placed centrally within the department and did not enter PUI rooms. Masking was mandated for all patients as well as staff in the ED, and masks were provided for those who did not have one. We also focused on PPE solutions, such as use of PAPR for staff comfort. While we had to employ PPE conservation strategies such as N95 mask reuse with UV sterilization between uses, the attention to correct PPE use allowed our staff to remain safe. (As of April 15, 2020, only three of 218 MD/APP/registered nurses tested positive for COVID-19 at one site.)

In terms of personal wellness, our department was instrumental in facilitating childcare for providers by partnering with volunteer medical students given interruptions in their educational schedule, and with professional childcare agencies after schools closed. Our department of EM also funded on-shift food for the EDs for two weeks at the beginning of increased COVID-19 PUI volume, and then transitioned to fundraising at an institutional level to continue to provide food for all ED staff on shift. Faculty also started collating personal locations to volunteer space (such as unused garage rooms or carriage houses) for those in need of quarantine or isolation outside their own homes, in addition to the hotel housing offered by our institution. Great attention was paid to the emotional state of providers and staff, with ongoing discussions normalizing and validating the range of emotions experienced and offering emotional processing groups at the end of weekly operations through virtual sessions led by department leadership. Counseling services were offered by phone or virtual platform by Emory’s Faculty and Staff Assistance Program and Emory Psychiatry. Yoga and meditation classes were offered on a virtual platform so employees could continue to participate while practicing social distancing.

#### Morale Recommendations

1. Implement PPE strategies that address both supply chain and staff morale, including universal masking to protect both patients and staff, offering alternative PPE solutions such as PAPR for staff comfort, and maintaining a strong PPE supply chain so that fear of lack of available PPE is reduced.
2. Support staff wellness through programming to address acute needs, such as childcare, quarantine housing, on-shift food, and emotional stress.

## CONCLUSION

**Figure 2:**
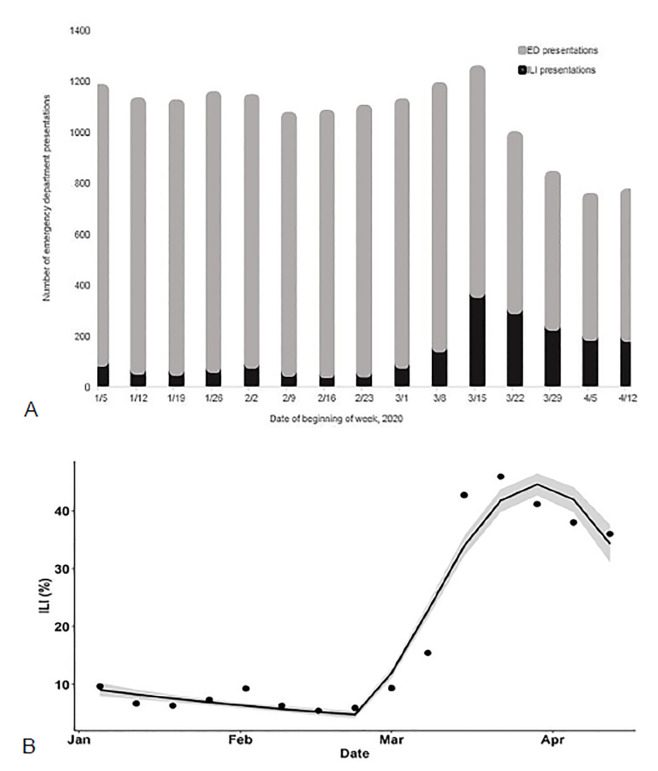
A) Weekly number of presentations, and presentations for Influenza-like illness (ILI) to the Emergency Department at Emory University Hospital during the COVID-19 pandemic in 2020. B) Broken-stick quadratic regression (95% CI) of weekly proportion of visits that were ILI with breakpoint set at the week beginning February 23 2020 (week of first announced death due to COVID-19 in the state of Washington) OR = 0.90, 95% CI: 0.88 – 0.91, p <.001.

These are just some of the interventions that we found to be helpful as our department learned to navigate this crisis. We are continuing to prepare, adapt, and innovate as we are faced with the changing realities of the COVID-19 pandemic each day and prepare for the transition between response and recovery, and back again. As with many healthcare systems around the US, we noticed an overall decline in ED volumes as well as an increase in influenza-like illness cases (Figures 2a, 2b) through March–April 2020. As our percentage of laboratory-confirmed influenza cases steadily decreased to zero, our laboratory confirmed COVID-19 cases peaked at 25.1% of those tested having a positive result during our initial surge (March 23–29, 2020). Our test positivity rate began steadily rising again in June–July 2020, greatly exceeding our initial surge. We are still struggling with adaptation to shifting guidelines and the unknowns of what is to come as individual governors allow stay-at-home orders to expire and with discordance in public masking recommendations.

While we initially disassembled our tents given reduced volumes, they remained on site and have been reconstructed given our new surge in COVID-19 patient volume. We are now experiencing increased ED boarding as inpatient beds are full with COVID-19 patients as well as postoperative patients after restarting elective surgery at our institution. The preparedness and processes put in place during the initial surge facilitated our team in adeptly managing patient care and ED flow as cases drastically increased. Without question, we will continue to use these lessons and recommendations on preparedness, adaptability, and innovation in this second surge of COVID-19 and in the future for inevitable additional waves, as well as for whatever emerging public health emergency comes next.
